# Protective Effects of Tropical Fruit Processing Coproducts on Probiotic *Lactobacillus* Strains during Freeze-Drying and Storage

**DOI:** 10.3390/microorganisms8010096

**Published:** 2020-01-10

**Authors:** Caroliny Mesquita Araújo, Karoliny Brito Sampaio, Francisca Nayara Dantas Duarte Menezes, Erika Tayse da Cruz Almeida, Marcos dos Santos Lima, Vanessa Bordin Viera, Estefânia Fernandes Garcia, Andrea Gómez-Zavaglia, Evandro Leite de Souza, Maria Elieidy Gomes de Oliveira

**Affiliations:** 1 Departamento de Nutrição, Centro de Ciências da Saúde, Universidade Federal da Paraíba, João Pessoa 58051-900, Brazil; carolnutripb@gmail.com (C.M.A.); karolbsampaio@gmail.com (K.B.S.); nutrinay@gmail.com (F.N.D.D.M.); erika_yse@hotmail.com (E.T.d.C.A.); elieidynutri@yahoo.com.br (M.E.G.d.O.); 2 Departamento de Tecnologia de Alimentos, Instituto Federal do Sertão de Pernambuco, Petrolina 56302-100, Brazil; marcos.santos@ifsertao-pe.edu.br; 3 Centro de Educação e Saúde, Universidade Federal de Campina Grande, Cuité 58175-000, Brazil; vanessa.bordinviera@gmail.com; 4 Departamento de Gastronomia, Centro de Tecnologia e Desenvolvimento Regional, Universidade Federal da Paraíba, João Pessoa 58058-600, Brazil; estefaniafgarcia@yahoo.com.br; 5 Center for Research and Development in Food Cryotechnology (CIDCA, CCT-CONICET), La Plata 1900, Argentina; angoza@qui.uc.pt

**Keywords:** fruit, agroindustrial coproducts, preservation, protectants, *Lactobacillus*, cell damage

## Abstract

This study evaluated the protective effects of coproducts from agroindustrial processing of the tropical fruits acerola (*Malpighia glabra* L., ACE), cashew (*Anacardium occidentale* L., CAS), and guava (*Psidium guayaba* L., GUA) on the probiotics *Lactobacillus paracasei* L-10, *Lactobacillus casei* L-26, and *Lactobacillus acidophilus* LA-05 during freeze-drying and storage. The occurrence of damage to membrane integrity, membrane potential, and efflux activity of *Lactobacillus* cells after freeze-drying was evaluated by flow cytometry, and viable counts were measured immediately after freeze-drying and during 90 days of storage under refrigerated or room temperature conditions. Probiotic strains freeze-dried without substrate had the overall highest count reductions (0.5 ± 0.1 to 2.9 ± 0.3 log cycles) after freeze-drying. Probiotics freeze-dried with fruit processing coproducts had small cell subpopulations with damaged efflux activity and membrane potential. Average counts of probiotics freeze-dried with ACE, CAS, or GUA after 90 days of storage under refrigerated or room temperature were in the range of 4.2 ± 0.1 to 5.3 ± 0.2 and 2.6 ± 0.3 to 4.9 ± 0.2 log CFU/g, respectively, which were higher than those observed for strains freeze-dried without substrate. The greatest protective effects on freeze-dried probiotics were overall presented by ACE. These results revealed that ACE, CAS, and GUA can exert protective effects and increase the stability of probiotic lactobacilli during freeze-drying and storage, in addition to supporting a possible added-value destination for these agroindustrial coproducts as vehicles for probiotics and for the development of novel functional foods.

## 1. Introduction

The consumption of fruit has increased worldwide due to the recognition of their nutritional value and because they are sources of compounds with health-related bioactive properties [1]. Brazil stands out in the production of tropical fruit, ranking the first in the world in the production of acerola (*Malpighia glabra* L., ACE), cashew (*Anacardium occidentale* L., CAS), and guava (*Psidium guayaba* L., GUA), which are fruits valued for their nutritional characteristics and good sensory acceptance [2].

Since fresh fruit are highly susceptible to decay, most fruit production is typically destined to the processing of juices and frozen pulps [3], and about 30–40% of the dry weight of fruit destined for juice and pulp processing is discarded as agroindustrial coproducts [4]. These fruit processing coproducts consist mainly of peels, seeds, and some mashed flesh, which are considered sources of compounds with different beneficial biological activities [5]. The consumption of acerola, cashew, and guava agroindustrial coproducts was reported to cause no damage to the histopathological characteristics of intestine and liver of rats, indicating the low potential toxicity of these materials [6].

Lactic acid bacteria (LAB) are biotechnological tools for different industry sectors that are used as starter cultures for food processing and functional food formulation. Among LAB, the *Lactobacillus* genus has been intensively studied for the selection of probiotic strains [7]. Health benefits derived from probiotic ingestion can generally be achieved when at least 6–7 log CFU of viable microorganisms per gram or milliliter of the carrier product are ingested [8].

Probiotic cultures are typically freeze-dried to produce powders with prolonged storage stability for delivery in food formulation or when ingested as food supplements [9]. Dehydration involved in freeze-drying process can cause structural damage (particularly during freezing) to bacterial cells, resulting in decreased viability and metabolic activity [10]. Alterations in the physical state of membrane lipids with the disturbance of their integrity and fluidity have been reported as the main cause for viability loss in freeze-dried probiotics [11].

Simple and complex matrices have been used as protectants to decrease the occurrence of damage and viability loss in probiotic cells during freeze-drying, such as skim milk, whey milk, amino acids, glycerol, sugars, and dietary fibers, including those with prebiotic properties (e.g., fructo-oligosaccharides, inulin, and raffinose). These ingredients act primarily by protecting the bacterial cultures from disturbing effects exerted by dehydration on the cell membrane [12,13,14]. Considering that fruit and their agroindustrial coproducts are sources of simple sugars and nondigestible carbohydrates (e.g., fiber and fructo-oligosaccharides) [5,6], it could be expected that these coproducts could protect probiotic cultures when subjected to dehydration processes, such as freeze-drying, representing a possible added-value use for these materials. Furthermore, the presence of different phenolic compounds with well-known antioxidant properties in fruit processing coproducts [6,15] could also help to protect bacterial cell membranes from oxidative damage commonly associated with dehydration processes [15,16,17].

Despite the increased interest in antioxidant and prebiotic components naturally present in fruit processing coproducts [5,18], studies on the protective effects of these materials on probiotic cells during exposure to dehydration processes are scarce. Only one previous study reported the stabilizing effects of okara, a coproduct from soy milk elaboration, on *Lactobacillus plantarum* during freeze-drying [19]. Considering these aspects, this study evaluated whether coproducts generated during the processing of acerola, cashew, and guava can exert protective effects on probiotic *Lactobacillus* strains during freeze-drying and storage.

## 2. Material and Methods

### 2.1. Preparation of Fruit Processing Coproducts

The processing coproducts of acerola (*Malpighia emarginata* D.C.), cashew (*A. occidentale* L.), and guava (*P. guayaba* L.) were obtained from three different fruit pulp processing companies located in the city of João Pessoa (Paraíba, Brazil). The samples (approximately 500 g each) were collected from four fruit processing batches from each company, which were pooled for a total of approximately 6 kg for each fruit species. Each type of fruit processing coproduct was separately packaged in heat-resistant plastic bags and then autoclaved (121 °C, 1 atm, 15 min) to eliminate contaminant microorganisms, frozen at −18 ± 2 °C for 24 h, and lyophilized using a benchtop freeze dryer (LI-101 model; LIOTOP^®^, São Carlos, Brazil) at −55 ± 2 °C, with a vacuum pressure of <138 μHG and a freeze-drying rate of <1 mm/h for approximately 12 h. The freeze-dried material was sieved through a fine mesh to obtain a powder of mean particle size <1 mm. The final product (powder) was stored at −18 ± 2 °C in hermetically sealed polypropylene bags.

### 2.2. Physicochemical Characterization of Fruit Processing Coproducts

The sugar (glucose, fructose, and maltose) and phenolic compound content profiles of the tested fruit processing coproducts were determined by high-performance liquid chromatography (HPLC) on an Agilent chromatograph (Infinity LC model 1260, Agilent Technologies, Santa Clara, CA, USA) using previously described analytical conditions [20,21]. Insoluble and soluble fiber contents were determined using an enzymatic–gravimetric method [22]. Total phenolic and flavonoid contents were determined according to procedures described elsewhere [23,24,25]. *In vitro* antioxidant activity of fruit coproducts was assessed by an iron reduction method (ferric reducing antioxidant power—FRAP) [26] and the ABTS method [27]. Values of the measured physicochemical parameters of acerola, cashew, and guava processing coproducts are presented in Table 1.

### 2.3. Evaluation of the Protective Effects of Fruit Processing Coproducts on Freeze-Dried Probiotic Lactobacillus Strains

#### 2.3.1. Microorganisms, Inoculum Preparation, and Treatments

Different well-known probiotic *Lactobacillus* strains, namely, *Lactobacillus acidophilus* LA-05, *Lactobacillus paracasei* L-10, and *Lactobacillus casei* L-26 [5,12,28], were used as test organisms. These strains were obtained from the Collection of Microorganisms, Faculty of Biotechnology, Catholic University of Porto (Porto, Portugal). Stock cultures were maintained in de Man, Rogosa, and Sharpe (MRS) broth (HiMedia, Mumbai, India) containing glycerol (150 g/L) at −18 ± 2 °C. For inoculum preparation, each *Lactobacillus* strain was firstly anaerobically cultured (Anaerogen System Anaerogen, Oxoid, Wade Road, Basingstoke, UK) in MRS broth at 37 °C up to reaching the stationary growth phase (20–24 h).

Cell mass was collected by centrifugation (4000× *g* for 10 min at 4 °C), washed twice in sterile saline (NaCl 8.5 g/L), and resuspended in five different treatments for each tested *Lactobacillus* strain, namely, (i) strain suspended in sterile distilled water (negative control, termed NEC); (ii) strain suspended in sterile distilled water with fructo-oligosaccharides (2% *w*/*v*, positive control, termed FOS); and (iii) three different suspensions of each strain in sterile distilled water with 2% (*w*/*v*) of ACE, CAS, or GUA processing coproduct (final pH ranging from 3.24 to 5.12). These suspensions provided viable counts varying from 9.1 to 10.3 log CFU/mL [with optical density (OD) reading at 625 nm (OD625) corresponding to 1.5]. The suspensions were transferred to capped glass vials (5 mL) under aseptic conditions and subjected to freezing at −80 °C for 24 h. The tested concentration of ACE, CAS, and GUA was selected based on the results of a previous study, which showed that 2% (*w*/*v*) of a tropical fruit processing coproduct positively affected the growth and metabolic activities of probiotic lactobacilli, indicating potential prebiotic effects [5].

#### 2.3.2. Freeze-Drying and Survival of Probiotic *Lactobacillus*

The frozen bacterial suspensions with ACE, CAS, or GUA as well as the frozen positive (FOS) and negative (NEC) controls were subjected to freeze-drying at −55 ± 2 °C, with a vacuum pressure of <138 μHG and a freeze-drying rate of 1 mm/h, for approximately 40 h using a benchtop freeze dryer (LIOTOP^®^, Model L-101, São Carlos-SP, Brazil). Just after freeze-drying, the viable cells of the tested strains were enumerated. For this, the freeze-dried strains in the different treatments were rehydrated in sterile distilled water (30 ± 0.5 °C) for 15 min. Serial dilutions were subsequently performed using sterile saline solution (NaCl 8.5 g/L) and dispensed onto MRS agar plates (HiMedia, Mumbai, India) using a microdrop inoculation technique [29]. After an incubation period of 48 h at 37 °C under anaerobic conditions (Anaerobic System Anaerogen, Oxoid), the visible colonies were enumerated and the results expressed as log CFU/g. The detection limit of the assays for viable cell counts was 1 log CFU/g.

#### 2.3.3. Evaluation of Damage to Membrane Functions of Probiotic *Lactobacillus* Cells after Freeze-Drying

Flow cytometry (FC) analysis was used to monitor the occurrence of damage to different membrane functions of the tested probiotic *Lactobacillus* strains in the absence (NEC) or presence of FOS, ACE, CAS, or GUA immediately after freeze-drying. Freeze-dried strains in the different treatments were rehydrated in sterile distilled water (30 ± 0.5 °C) for 15 min and centrifuged (4500× *g* for 10 min at 4 °C). The obtained pellets were washed twice and resuspended in sterile phosphate-buffered saline (PBS; 8.0 g/L of NaCl, 0.20 g/L of KCl, 1.44 g/L of Na_2_HPO_4_, and 0.24 g/L of KH_2_PO_4_; pH 7.4) and immediately labeled with propidium iodide (PI; Sigma-Aldrich, St. Louis, MO, USA) to evaluate membrane integrity; trimetine oxonol of bis-(1,3-dibutylbarbituric acid) [(DiBAC_4_(3), or BOX; Molecular Probes, Invitrogen, OR, USA] to evaluate membrane potential; and ethidium bromide (EB; Sigma-Aldrich, St. Louis, MO, USA) to evaluate efflux activity, following previously described cell staining procedures [30,31,32].

FC measurements were performed using a flow cytometer equipped with an argon ion laser at 488 nm (BD Accuri C6, Piscataway, NJ, USA). Green and red fluorescence were collected on FL1 (533 ± 30 nm) and FL3 (>670 nm) channels, respectively. Dispersion and fluorescence signals from individual cells passing through the laser zone were collected as logarithmic signals. The fluorescence signal (pulse area measurements) was collected by FL1 [DiBAC_4_(3)] and FL3 (PI and EB) band filters. The threshold level for data acquisition was defined for FSC (12,000) in order to eliminate the background and signs of debris considered to be much smaller than the intact bacterium. Bacteria cells were identified by FSC/SSC parameters. Each sample acquisition was operated at a low flow rate and a total of 10,000 events were analyzed. All fluorescence emission cytograms were recorded using the BD Accuri C6 Software (BD^®^, Becton Dickinson and Company, Franklin Lakes, NJ, USA).

SSC density plot analysis versus FL1 or FL3 was applied to determine the fluorescence properties of the PI^+^, DiBAC_4_(3)^+^, and EB^+^ populations, respectively, indicating cells with a damaged membrane, depolarized membrane, and altered efflux pump, respectively, which had their populations identified through rectangles located on the right side of the graphs.

#### 2.3.4. Enumeration of Viable Cells of Freeze-Dried Probiotic *Lactobacillus* during Storage

The probiotic strains freeze-dried (counts of approximately 7–10 log CFU/g) in the absence (NEC) or presence of the different substrates (FOS, ACE, CAS, and GUA) were stored in sealed vials, which were maintained in desiccators containing silica gel for relative humidity control and stored under refrigerated (4 ± 0.5 °C) or room temperature (25 ± 0.5 °C) conditions for 90 days. At regular storage time intervals of 15 days, the strains freeze-dried without or with substrates were rehydrated in sterile distilled water (1:9 ratio) at room temperature (25 ± 0.5 °C), serially diluted using sterile saline solution (NaCl 8.5 g/L), and inoculated onto MRS agar plates using a microdrop inoculation technique [27]. After an incubation period of 48 h at 37 °C under anaerobic conditions (Anaerobic System Anaerogen, Oxoid), the visible colonies were enumerated and the results expressed as log CFU/g. The detection limit of the assays for viable cell counts was 1 log CFU/g.

### 2.4. Statistical Analysis

The assays were performed in triplicate in two independent experiments and the results were expressed as average ± standard deviation. Data were submitted to Student’s *t* test or analysis of variance (ANOVA) followed by Tukey’s test using *p* ≤ 0.05. Graphpad Prism 6.0 (GraphPad Software Inc., San Diego, CA, USA) was used to perform statistical analyses. FC analyses were performed in duplicate in two independent experiments with consistent results.

## 3. Results and Discussion

### 3.1. Viable Counts of Probiotic Lactobacillus before and after Freeze-Drying

The suspensions of the tested probiotic *Lactobacillus* strains without or with the tested substrates separately had viable counts (counts) in the range of 9.3 ± 0.1 to 10.3 ± 0.2 and 9.1 ± 0.2 to 10.3 ± 0.3 log CFU/g before freeze-drying, respectively. After freeze-drying, the counts of *L. casei* L-26 and *L. acidophilus* LA-05 without or with FOS, ACE, CAS, or GUA decreased (*p* ≤ 0.05), but this behavior was not observed for *L. paracasei* L-10 freeze-dried with FOS or ACE (*p* > 0.05). The highest average log reductions after freeze-drying (0.5 ± 0.1 to 2.9 ± 0.3 log CFU/g) were in most cases observed when the strains were freeze-dried without substrate (NEC), especially for *L. casei* L-26, which presented average count reductions of approximately 2.9 ± 0.3 log CFU/g. The average log reductions for the tested *Lactobacillus* strains freeze-dried with ACE, CAS, or GUA were in the range of 0.2 ± 0.0 to 2.0 ± 0.3 log cycles (Table 2). The decreasing rank of average log reduction after freeze-drying for the tested probiotic *Lactobacillus* strains, regardless of the treatment, was *L. casei* L-26 > *L. acidophilus* LA-05 > *L. paracasei* L-10, indicating that the protective effects exerted by ACE, CAS, and GUA should be strain-dependent. Conversely, the available literature has reported that protective effects on bacterial cultures during dehydration processes should not depend only on the physicochemical characteristics of protectant compounds but also on the intrinsic sensitivity of the tested strains [12,13,14].

These results suggest that ACE, CAS, and GUA could act as protectants with, in most cases, similar efficacy when compared to FOS (composed only of sugars and widely known because of its protective effects [12,13]), decreasing potential negative impacts on the viability of tested probiotic *Lactobacillus* during freeze-drying.

### 3.2. Evaluation of Damage to Membrane Functions of Probiotic Lactobacillus Cells after Freeze-Drying

The probiotic *Lactobacillus* strains freeze-dried without (NEC) or with FOS, ACE, CAS, or GUA were analyzed by FC using three fluorescent probes [PI, EB, and DIBAC_4_(3)] separately to verify if the tested fruit processing coproducts may decrease the damage commonly caused by freeze-drying to the bacterial membrane.

**Figure 1:**
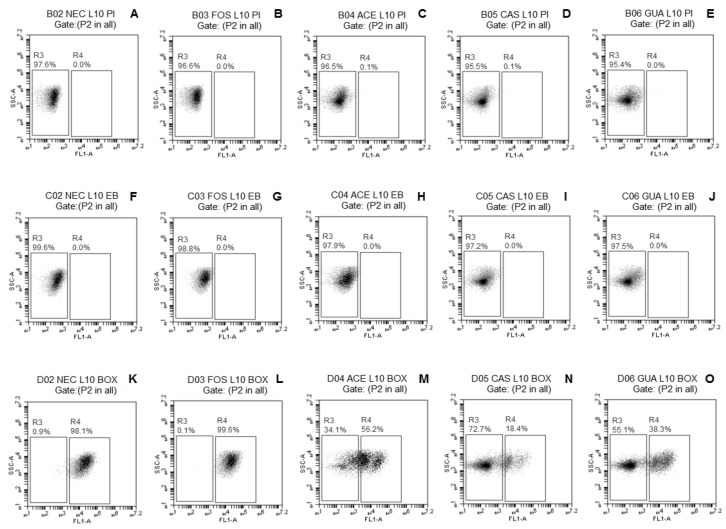
Fluorescence density plots of *L. paracasei* L-10 in response to staining with propidium iodide (PI) (**A**–**E**), ethidium bromide (EB) (**F**–**J**), and biz-1,3-dibutylbarbaturic acid [DiBAC_4_(3)] (**K**–**O**) after freeze-drying in the absence (NEC, ●) or presence of fructo-oligosaccharides (FOS, ■) or processing coproducts of acerola (ACE, □), cashew (CAS, ▵), or guava (GUA, ○). L10—*L. paracasei* L-10. The vertical axis indicates the fluorescence intensity of PI, EB, and DiBAC_4_(3) and the intensity of lateral light dispersal. The subpopulation of negative stains was blocked in the left rectangles; the positive subpopulation of the stains was delimited in the right rectangles. The percentages of cell subpopulations that fell on each port are shown in each plot.

**Figure 2:**
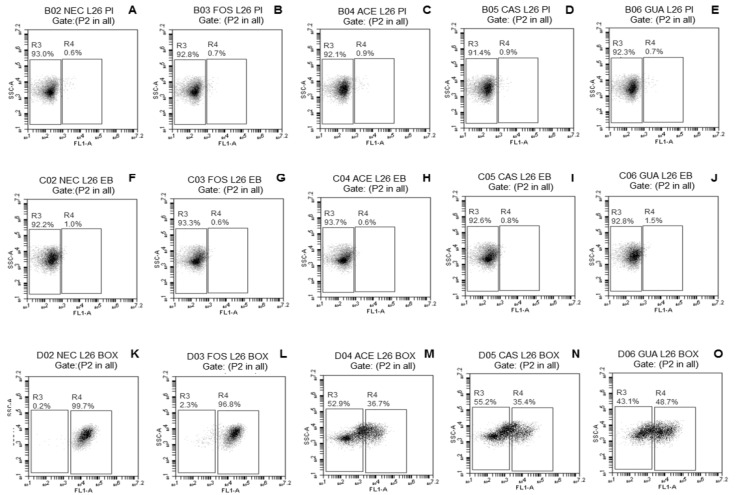
Fluorescence density plots of *L. casei* L-26 in response to staining with PI (**A**–**E**), EB (**F**–**J**), and DiBAC_4_(3) (**K**–**O**) after freeze-drying in the absence (NEC, ●) or presence of fructo-oligosaccharides (FOS, ■) or processing coproducts of acerola (ACE, □), cashew (CAS, ▵), or guava (GUA,○). L26—*L. casei* L-26. The vertical axis indicates the fluorescence intensity of PI, EB, and DiBAC_4_(3) and the intensity of lateral light dispersal. The subpopulation of negative stains was blocked in the left rectangles; the positive subpopulation of the stains was delimited in the right rectangles. The percentages of cell subpopulations that fell on each port are shown in each plot.

**Figure 3:**
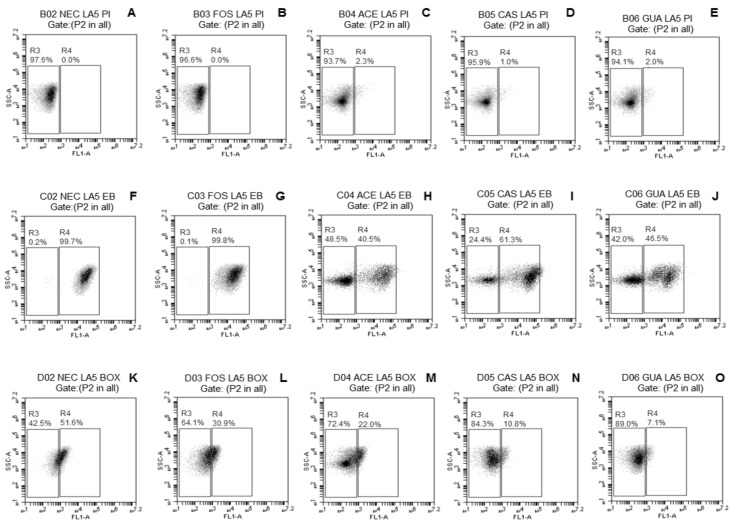
Fluorescence density plots of *L. acidophilus* LA-05 in response to staining with PI (**A**–**E**), EB (**F**–**J**), and DiBAC_4_(3) (**K**–**O**) after freeze-drying in the absence (NEC, ●) or presence of fructo-oligosaccharides (FOS, ■) or processing coproducts of acerola (ACE, □), cashew (CAS, ▵), or guava (GUA, ○). LA-05—*L. acidophilus* LA-05. The vertical axis indicates the fluorescence intensity of PI, EB, and DiBAC_4_(3) and the intensity of lateral light dispersal. The subpopulation of negative stains was blocked in the left rectangles; the positive subpopulation of the stains was delimited in the right rectangles. The percentages of cell subpopulations that fell on each port are shown in each plot.

The fluorescence density plots of *L. paracasei* L-10 (Figure 1A–E) demonstrated no evident change in membrane integrity of this strain when freeze-dried without or with the different tested substrates (only 0.1% subpopulation with damage when freeze-dried with ACE or CAS). A small subpopulation of *L. casei* L-26 cells (<1%) (Figure 2A–E) with damaged membrane integrity was observed regardless of the use of substrates during freeze-drying. No damage to membrane integrity was observed in *L. acidophilus* LA-05 cells (Figure 3A–E) freeze-dried without substrate (NEC) or with FOS. Although subpopulations of cells with damaged membrane integrity were observed for *L. acidophilus* LA-05 freeze-dried with ACE, CAS, or GUA, the sizes of these subpopulations were always very small (1–2.3%).

The fluorescence density plots of *L. paracasei* L-10 (Figure 1F–J) showed no damage to membrane efflux activity when this strain was freeze-dried without or with the different tested substrates. *L. casei* L-26 (Figure 2F–J) showed a small subpopulation of cells with damaged efflux activity when freeze-dried without (1%) or with ACE, CAS, GUA, or FOS (0.6–1.5%). *L. acidophilus* LA-05 (Figure 3F–J) was the strain with the highest cell subpopulations with damaged efflux activity when freeze-dried without or with any of the tested substrates. However, the subpopulations of *L. acidophilus* LA-05 cells with damaged efflux activity were smaller when this strain was freeze-dried with ACE, CAS, or GUA (40.5–61.3%) when compared with freeze-drying without substrate (NEC, 99.7%) or with FOS (99.8%).

Higher subpopulations of *L. paracasei* L-10 cells with membrane depolarization (Figure 1K–O) were observed when the strain was freeze-dried without substrate (NEC, 98.1%) or with FOS (99.6%) when compared with freeze-drying with ACE (56.2%), CAS (18.4%), or GUA (38.3%). Similarly, smaller subpopulations of *L. casei* L-26 and *L. acidophilus* LA-05 cells with depolarized membranes were observed when these strains were freeze-dried with ACE (36.7% and 22.0%, respectively), CAS (35.4% and 10.8%, respectively), and GUA (48.7% and 7.1%, respectively) than when freeze-dried without substrate (NEC, 99.7% and 51.6%, respectively) or with FOS (96.8% and 30.9%, respectively) (Figure 2K–O and Figure 3K–O, respectively).

*L. casei* L-26 presented the highest cell subpopulations with damage to membrane potential after freeze-drying with tested fruit processing coproducts, while *L. acidophilus* LA-05 presented the highest cell subpopulations with damage to efflux activity, although the latter also presented cell subpopulations with damage to membrane potential. Otherwise, *L. paracasei* L-10 presented overall low cell subpopulations with damage to membrane potential and efflux activity after freeze-drying with tested fruit processing coproducts. These results are overall consistent with the results of the assays to measure the viable count reductions after freeze-drying, where *L. paracasei* L-10 had lower viable count reductions compared with *L. acidophilus* LA-05 and *L. casei* L-26.

The physicochemical characteristics (Table 1) of fruit processing coproducts used during the freeze-drying of the tested probiotic *Lactobacillus* strains, which showed considerable amounts of simple sugars (e.g., glucose, fructose, and maltose) and fiber (particularly insoluble fiber) contents, could enable these materials to protect the cell membranes of these microorganisms, decreasing the damage that typically occurs during freeze-drying. Monosaccharides and disaccharides normally have a low glass transition temperature (*T_g_*) and exert an efficient protecting effect on bacteria during freeze-drying, since they are able to interact with the lipids of bacterial membranes and replace water molecules [33]. Polysaccharides and oligosaccharides, which exhibit high *T_g_*, are usually less effective than monosaccharides at protecting bacterial cells during freeze-drying; however, because polysaccharides can favor the formation of vitreous states, they may also exert some stabilizing effects on bacterial cells during freeze-drying [13].

The bioactive compounds of tropical fruit have shown antioxidant and free-radical-sequestering properties, which can slow or inhibit the oxidative damage of proteins and lipids in cells [15,34]. The high amounts of phenolic compounds and total flavonoids in the tested fruit processing coproducts, mainly in ACE, possibly contributed to their higher antioxidant activities (as observed in FRAP or ABTS assays, Table 1). These characteristics may be also associated with the protection of cell membranes observed for *Lactobacillus* strains freeze-dried with the tested fruit processing coproducts, especially with ACE, since dehydration processes can render bacterial cells susceptible to reactive oxygen species that can directly attack polyunsaturated fatty acids in membranes and disturb membrane-bound proteins, affecting the structure and function of membranes in bacterial cells [16,17].

### 3.3. Viable Counts of Freeze-Dried Lactobacillus during Storage

**Figure 4:**
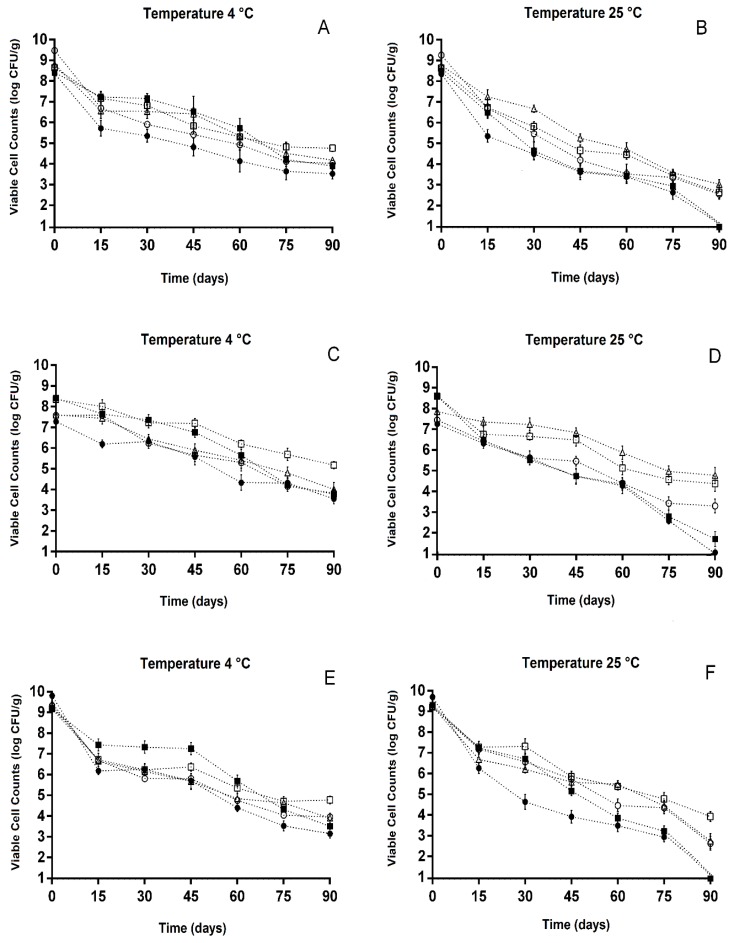
Viable cell counts (log CFU/g) of (**A**) *L. paracasei* L-10, (**B**) *L. casei* L-26, and (**C**) *L. acidophilus* LA-05 freeze-dried in the absence (NEC, ●) or presence of fructo-oligosaccharides (FOS, ■) or processing coproducts of acerola (ACE, □), cashew (CAS, ▵), or guava (GUA, ○) during 90 days of storage at 4 ± 0.5 °C (**A**,**C**,**E**) or 25 ± 0.5 °C (**B**,**D**,**F**).

The viable counts of freeze-dried *L. paracasei* L-10 (Figure 4A,B), *L. casei* L-26 (Figure 4C,D), and *L. acidophilus* LA-05 (Figure 4E,F) were evaluated during 90 days of storage under refrigerated (4 ± 0.5 °C) and room temperature (25 ± 0.5 °C) conditions. The freeze-dried *Lactobacillus* strains presented viable counts in the range of 3.2 ± 0.1 to 9.9 ± 0.1 log CFU/g over the 90 days of storage under refrigeration. The viable counts of tested *Lactobacillus* strains freeze-dried with ACE, CAS, or GUA (4.2 ± 0.1 to 5.3 ± 0.2 log CFU/g) at the end of the 90 days of storage were higher (*p* ≤ 0.05) when stored under refrigeration than under room temperature (2.6 ± 0.2 to 4.9 ± 0.2 log CFU/g). However, the viable counts of tested *Lactobacillus* strains freeze-dried with ACE, CAS, or GUA (2.6 ± 0.3 to 4.9 ± 0.2 log CFU/g) at the end of the 90 days of room temperature storage were higher (*p* ≤ 0.05) than those observed when these strains were freeze-dried without substrate (NEC, 1.1 ± 0.3 log CFU/g) or with FOS (1.2 ± 0.1 to 1.8 ± 0.3 log CFU/g).

*L. paracasei* L-10 (Figure 4A,B) freeze-dried without (NEC) or with FOS, ACE, CAS, or GUA showed sharp reductions in viable counts after 15 days of storage under refrigerated or room temperature conditions, especially when this strain was freeze-dried without substrate (NEC, log reductions of approximately 2.7 ± 0.2). The viable counts of *L. paracasei* L-10 decreased during the 90 days of storage under refrigeration regardless of the use of substrate during freeze-drying (Figure 4A), but the highest decreases (reduction > 5 log CFU/g) were observed when this strain was freeze-dried without substrate (NEC). The viable counts of *L. paracasei* L-10 after 90 days of refrigerated storage were higher (*p* ≤ 0.05) when this strain was freeze-dried with ACE (4.8 ± 0.2 log CFU/g) compared with freeze-drying with CAS (4.2 ± 0.3 log CFU/g) or GUA (4.2 ± 0.2 log CFU/g); however, at this same storage time point, the viable counts of *L. paracasei* L-10 freeze-dried with ACE, CAS, or GUA were higher (*p* ≤ 0.05) compared with the freeze-drying without substrate (NEC, 3.5 ± 0.2 log CFU/g) or with FOS (3.7 ± 0.2 log CFU/g).

A small decrease (<1 log CFU/g) in viable counts of *L. casei* L-26 (Figure 4C,D) was observed after 15 days of storage under refrigeration when the strain was freeze-dried with ACE, CAS, GUA, or FOS; at this same storage time point, *L. casei* L-26 freeze-dried without substrate (NEC) showed a decrease in viable counts of >1 log CFU/g (Figure 4C). The viable counts of *L. casei* L-26 freeze-dried without or with substrates overall decreased from 15 days of refrigerated storage onward. The viable counts of *L. casei* L-26 freeze-dried with ACE (5.3 ± 0.3 log CFU/g) were higher (*p* ≤ 0.05) than those observed when this strain was freeze-dried with CAS (4.2 ± 0.3 log CFU/g) or GUA (4.1 ± 0.1 log CFU/g) after 90 days of storage under refrigeration.

The viable counts of *L. acidophilus* LA-05 decreased (*p* ≤ 0.05) after 15 days of refrigerated or room temperature storage regardless of the use of substrate during freeze-drying (Figure 4E,F); however, the counts of this strain when freeze-dried with ACE or FOS did not change (*p* > 0.05) from 15 to 45 days of refrigerated storage. The viable counts of *L. acidophilus* LA-05 at the end of the 90 days of refrigerated storage were higher when this strain was freeze-dried with ACE (4.7 ± 0.2 log CFU/g) in comparison with freeze-drying with CAS (4.2 ± 0.3 log CFU/g), GUA (4.1 ± 0.2 log CFU/g), or FOS (3.7 ± 0.3 log CFU/g) or without substrate (NEC) (3.3 ± 0.2 log CFU/g).

Freeze-dried cultures should also be capable of tolerating storage at temperatures higher than those used in refrigeration [35]. However, when stored under room temperature (i.e., 22–25 °C), the decreases in viable counts of dehydrated cultures may be up to 10-fold higher than those observed under refrigerated storage [36]. This evidence reinforces the data observed in the present study, which showed a more pronounced drop in the viable counts of the tested probiotic *Lactobacillus* strains when stored under room temperature, indicating that refrigerated storage of *Lactobacillus* strains freeze-dried with ACE, CAS, or GUA could be the most appropriate. Further investigations should be carried out to optimize the freeze-drying conditions of probiotic *Lactobacillus* in the presence of ACE, CAS, and GUA (i.e., in combination with other protectants and considering variations in the amounts of the tested fruit processing coproducts and length of freeze-drying period), as well as the storage conditions (mainly moisture) in order to measure their possible impacts on the observed protective effects.

## 4. Conclusions

ACE, CAS, and GUA processing coproducts exerted protective effects on probiotic *Lactobacillus* strains when subjected to freeze-drying and prolonged storage under refrigerated and room temperature conditions. Higher protective effects were observed when the *Lactobacillus* strains were freeze-dried with fruit processing coproducts and stored under refrigeration. Strains freeze-dried with ACE, CAS, or GUA presented decreased damage to efflux activity and membrane potential, especially when freeze-dried with ACE, indicating greater protection of bacterial cells from lesions in membranes caused by freeze-drying. Altogether, the presence of sugars, fibers, and phenolic compounds in ACE, CAS, and GUA could be directly associated with their stabilizing effects on the tested probiotics during freeze-drying and storage. These results overall demonstrate that ACE, CAS, and GUA agroindustrial processing coproducts have the potential to be exploited as innovative substrates to protect and increase the stability of probiotic *Lactobacillus* during freeze-drying and storage. Additionally, these results support a possible added-value destination for these materials as vehicles for probiotics and for the development of novel functional foods, decreasing the potential negative impacts on the environment related to the inadequate disposal of agroindustrial processing coproducts.

## Figures and Tables

**Table 1:** Physicochemical characteristics (mean ± standard deviation) of freeze-dried fruit processing coproducts used in assays of protective effect on probiotic *Lactobacillus* strains.

Parameters	Fruit Processing Coproducts		
	Acerola (ACE)	Cashew (CAS)	Guava (GUA)
Simple sugars (g/100 g)			
Fructose	8.48 ± 0.01 ^a^	4.80 ± 0.01 ^b^	3.92 ± 0.01 ^c^
Glucose	5.31 ± 0.01 ^a^	4.88 ± 0.01 ^b^	3.17 ± 0.01 ^c^
Maltose	1.52 ± 0.01 ^b^	1.97 ± 0.01 ^a^	1.53 ± 0.01 ^b^
Dietary fiber (g/100 g)			
Insoluble dietary fiber	61.16 ± 1.75 ^a^	47.49 ± 2.26 ^b^	49.12 ± 1.58 ^b^
Soluble dietary fiber	8.09 ± 0.69 ^b^	1.74 ± 0.53 ^c^	33.44 ± 3.63 ^a^
Total dietary fiber	69.25 ± 1.06 ^b^	49.22 ± 1.73 ^c^	82.55 ± 2.05 ^a^
Phenolic compounds (mg/100 g)			
Flavanols			
Catechin	3.12 ± 0.00	ND	1.95 ± 0.02
Flavanones			
Hesperetin	1.43 ± 0.01 ^b^	1.25 ± 0.01 ^c^	1.61 ± 0.01 ^a^
Naringenin	1.37 ± 0.01 ^a^	0.42 ± 0.01 ^b^	0.31 ± 0.01 ^c^
Flavonols			
Kaempferol	1.18 ± 0.01 ^a^	0.50 ± 0.02 ^c^	0.81 ± 0.02 ^b^
Myricetin	0.49 ± 0.01 ^c^	2.71 ± 0.06 ^a^	0.84 ± 0.00 ^b^
Quercitin	4.16 ± 0.01 ^a^	0.91 ± 0.02 ^b^	0.89 ± 0.03 ^b^
Rutin	1.19 ± 0.01	0.97 ± 0.02	ND
Hydroxybenzoic acids			
Syringic acid	ND	0.91 ± 0.07	0.52 ± 0.03
Hydroxycinnamic acids			
Caffeic acid	0.56 ± 0.01 ^b^	0.55 ± 0.01 ^b^	1.21 ± 0.01 ^a^
p-Coumaric acid	0.39 ± 0.01	ND	ND
Caftaric acid	0.92 ± 0.01 ^b^	1.32 ± 0.01 ^a^	0.64 ± 0.01 ^c^
Chlorogenic acid	0.35 ± 0.01 ^b^	0.31 ± 0.01 ^b^	0.62 ± 0.03 ^a^
Polyphenols			
Trans-resveratrol	1.12 ± 0.02 ^a^	0.45 ± 0.01 ^b^	0.32 ± 0.01 ^c^
Cis-resveratrol	1.51 ± 0.07 ^a^	0.27 ± 0.01 ^c^	0.91 ± 0.05 ^b^
Epicatechin gallate	0.37 ± 0.01 ^c^	0.71 ± 0.01 ^b^	1.22 ± 0.02 ^a^
Epicatechin	ND	1.04 ± 0.05	1.25 ± 0.05
Anthocyanins			
Petunidin 3-glucoside	0.49 ± 0.01	1.25 ± 0.05	ND
Pelargonidin 3-glucoside	ND	1.11 ± 0.01	ND
Procyanidin B1	ND	0.62 ± 0.04	0.51 ± 0.01
Procyanidin B2	ND	1.69 ± 0.09	0.43 ± 0.01
Procyanidin A2	ND	1.05 ± 0.01	1.13 ± 0.01
Total flavonoids (mg EC/100 g) ^1^	79.83 ± 0.23 ^a^	44.49 ± 0.61 ^b^	44.09 ± 1.01 ^b^
Total phenolics (mg EAG/100 g) ^2^	492.107 ± 0.54 ^a^	368.520 ± 1.09 ^b^	304.057 ± 0.94 ^c^
FRAP (µmol TEAC/g) ^3^	0.92 ± 0.01 ^a^	0.88 ± 0.01 ^b^	0.74 ± 0.01 ^c^
ABTS (µmol TEAC/g) ^3^	16.14 ± 0.01 ^a^	15.29 ± 0.01 ^b^	14.54 ± 0.01 ^c^

^a–c^ Mean ± standard deviation with different lowercase letters on the same row differ (*p* ≤ 0.05) among coproducts, based on Tukey’s test. ^1^ The results are expressed in milligram equivalents of catechin (EC) per hundred grams of sample (mg EC/100 g). ^2^ The results are expressed in milligram equivalents of gallic acid (EAG) per hundred grams of sample (mg EAG/100 g). ^3^ The results are expressed as micromoles of Trolox equivalent antioxidant capacity (TEAC) per gram of sample (µmol TEAC/g). Abbreviations: ND—not detected; FRAP—ferric reducing antioxidant power.

**Table 2:** Viable cell counts (log CFU/g) of probiotic *Lactobacillus* strains before and after freeze-drying without or with fructo-oligosaccharides, acerola, cashew, and guava processing coproducts.

Treatments	Strains								
	*Lactobacillus paracasei* L-10			*Lactobacillus casei* L-26			*Lactobacillus acidophilus* LA-05		
	Before Freeze-Drying	After Freeze-Drying	Average log Reduction *	Before Freeze-Drying	After Freeze-Drying	Average log Reduction *	Before Freeze-Drying	After Freeze-Drying	Average log Reduction *
NEC	9.3 ± 0.1 **	8.7 ± 0.2	0.5 ± 0.1 ^aC^	10.2 ± 0.1 **	7.3 ± 0.4	2.9 ± 0.3 ^aA^	10.3 ± 0.2 **	9.4 ± 0.1	0.9 ± 0.1 ^aB^
FOS	9.1 ± 0.2	8.9 ± 0.1	0.2 ± 0.0 ^bC^	10.2 ± 0.1 **	8.9 ± 0.3	1.3 ± 0.1 ^bA^	10.3 ± 0.1 **	9.4 ± 0.2	0.8 ± 0.0 ^aB^
ACE	9.1 ± 0.2	9.0 ± 0.2	0.2 ± 0.1 ^bC^	10.3 ± 0.1 **	8.9 ± 0.2	1.4 ± 0.3 ^bA^	10.2 ± 0.2 **	9.3 ± 0.1	0.9 ± 0.1 ^aB^
CAS	9.5 ± 0.2 **	8.9 ± 0.1	0.6 ± 0.1 ^aC^	9.8 ± 0.1 **	7.9 ± 0.4	2.0 ± 0.3 ^bA^	10.2 ± 0.2 **	9.2 ± 0.1	1.0 ± 0.1 ^aB^
GUA	9.3 ± 0.1 **	9.1 ± 0.1	0.3 ± 0.0 ^bC^	9.4 ± 0.1 **	7.6 ± 0.4	1.8 ± 0.3 ^bA^	10.2 ± 0.1 **	9.3 ± 0.2	1.0 ± 0.1 ^aB^

NEC: freeze-drying without fruit coproducts (negative control); FOS: freeze-drying with fructo-oligosaccharides (positive control); ACE: freeze-drying with acerola processing coproduct; CAS: freeze-drying with cashew processing coproduct; GUA: freeze-drying with guava processing coproduct. * Calculated from the difference of the viable counts (CFU/g) of the tested strain before and after freeze-drying. ** Denote significant differences (*p* ≤ 0.05) among the average counts before and after freeze-drying of the same strain, based on Student’s *t* test. ^a,b:^ Different superscript small letters at a column, for a same strain, denote significant differences (*p* ≤ 0.05) of average log reduction among different treatments, based on Tukey’s test. ^A–C:^ Different superscript capital letters at a row, for different strains, denote significant differences (*p* ≤ 0.05) of average log reduction for the same treatment, based on Tukey’s test.

## References

[1] Gonzalez-Aguilar G., Villa-Rodriguez J., Ayala-Zavala J., Yahia E. (2010). Improvement of the antioxidant status of tropical fruits as a secondary response to some postharvest treatments. Trends Food Sci. Technol..

[2] Silva L.M.R., Figueiredo E.A.T., Ricardo N.M.P.S., Vieira I.G.P., Figueiredo R.W., Brasil I.M., Gomes C.L. (2014). Quantification of bioactive compounds in pulps and by-products of tropical fruits from Brazil. Food Chem..

[3] Infante J., Selani M.M., Toledo N.M.V., Silveira-Diniz M.F., Alencar S.M., Spoto M.H.F. (2013). Antioxidant activity of agroindustrial residues from tropical fruits. Braz. J. Food Nutr..

[4] Nóbrega E.M., Oliveira E.L., Genovese M.I., Correia R.T.P. (2015). The Impact of Hot Air Drying on the Physical-Chemical Characteristics, Bioactive Compounds and Antioxidant Activity of Acerola (*Malphigia emarginata*) Residue. J. Food Process. Preserv..

[5] Duarte F.N.D., Rodrigues J.B., Lima M.C., Lima M.S., Pacheco M.T.B., Pintado M.M.E., Aquino J.S., Souza E.L. (2017). Potential prebiotic properties of cashew apple (*Anacardium occidentale* L.) agro-industrial byproduct on *Lactobacillus* sp.. J. Sci. Food Agric..

[6] Batista K.S., Alves A.F., Lima M.D.S., Silva L.A., Lins P.P., Gomes J.A.S., Silva A.S., Toscano L.T., Meireles B.R.L.A., Cordeiro A.M.T.M. (2018). Beneficial effects of consumption of acerola, cashew or guava processing by-products on intestinal health and lipid metabolism in dyslipidaemic female Wistar rats. Br. J. Nutr..

[7] Sun Z., Harris H.M.B., McCann A., Guo C., Argimón S., Zhang W., Yang X., Jeffery I.B., Cooney J.C., Kagawa T.F. (2015). Expanding the biotechnology potential of *lactobacilli* through comparative genomics of 213 strains and associated genera. Nat. Commun..

[8] Hill C., Guarner F., Reid G., Gibson G.R., Merenstein D.J., Pot B., Morrellj L., Canani R.B., Flint H.J., Salminen S. (2014). Expert consensus document: The International Scientific Association for Probiotics and Prebiotics consensus statement on the scope and appropriate use of the term probiotic. Nat. Rev. Gastroenterol. Hepatol..

[9] Garcia E.F., Araújo A.O., Luciano W.A., Albuquerque T.M.R., Arcanjo N.M.O., Madruga M.S., Lima M.S., Saarela M., Souza E.L. (2018). The performance of five fruit-derived and freeze-dried potentially probiotic *Lactobacillus* strains in apple, orange, and grape juices. J. Sci. Food Agric..

[10] Tymczyszyn E.E., Díaz M.R., Pataro A., Sandonato N., Gómez-Zavaglia A., Disalvo E.A. (2008). Critical water activity for the preservation of *Lactobacillus bulgaricus* by vacuum drying. Int. J. Food Microbiol..

[11] Velly H., Bouix M., Passot S., Penicaud C., Beinsteiner H., Ghorbal S., Lieben P., Fonseca F. (2015). Cyclopropanation of unsaturated fatty acids and membrane rigidification improve the freeze-drying resistance of *Lactococcus lactis* subsp. *Lactis* TOMSC161. Appl. Microbiol. Biotechnol..

[12] Nunes G.L., Etchepare M.A., Cichoski A.J., Zepka L.Q., Lopes E.J., Barin J.S., Flores E.M.M., Silva C.B., Menezes C.R. (2018). Inulin, hi-maize, and trehalose as thermal protectants for increasing viability of *Lactobacillus acidophilus* encapsulated by spray drying. LWT-Food Sci. Technol..

[13] Romano N., Schebor C., Mobili P., Gómez-Zavaglia A. (2016). Role of mono- and oligosaccharides from FOS as stabilizing agents during freeze-drying and storage of *Lactobacillus delbrueckii* subsp. bulgaricus. Food Res. Int..

[14] Tymczyszyn E.E., Gerbino E., Illanes A., Gomez-Zavaglia A. (2011). Galacto-oligosaccharides as protective molecules in the preservation of *Lactobacillus delbrueckii* subsp. bulgaricus. Cryobiology.

[15] Ayala-Zavala J.F., Vega-Vega V., Rosas-Dominguez C., Palafox-Carlos H., Villa-Rodriguez J.A., Siddiqui M. (2011). Agro-industrial potential of exotic fruit byproducts as a source of food additives. Food Res. Int..

[16] Leslie S.B., Israeli E., Lighthart B., Crowe J.H., Crowe L.M. (1995). Trehalose and sucrose protect both membranes and proteins in intact bacteria during drying. Appl. Environ. Microbiol..

[17] Teixeira P., Castro H., Kirby R. (1996). Evidence of membrane lipid oxidation of spray-dried *Lactobacillus bulgaricus* during storage. Lett. Appl. Microbiol..

[18] Liu X., Yan X., Bi J., Liu J., Zhou M., Wu X., Chen Q. (2018). Determination of phenolic compounds and antioxidant activities from peel, flesh and seed of guava (*Psidium guajava* L.). Electrophoresis.

[19] Quintana G., Gerbino E., Gómez-Zavaglia A. (2017). Okara: A nutritionally valuable by-product able to stabilize *Lactobacillus plantarum* during freeze-drying, spray-drying, and storage. Front. Microbiol..

[20] Ball S., Bullock S., Lloyd L., Mapp K.P., Ewen A. (2011). Analysis of carbohydrates, alcohols, and organic acids by ion-exchange chromatography. Agilent Hi-Plex Columns Applications Compendium.

[21] Padilha C.V.S., Miskinis G.A., Souza M.E.A.O., Pereira G.E., Oliveira D., Bordignon-Luiz M.T., Lima M.S. (2017). Rapid determination of flavonoids and phenolic acids in grape juices and wines by RP-HPLC/DAD: Method validation and characterization of commercial products of the new Brazilian varieties of grape. Food Chem..

[22] Horwitz W., Chichilo P., Reynolds H. (2010). Official Methods of Analysis of the Association of Official Analytical Chemists.

[23] Liu M., Li X.Q., Weber C., Lee C.Y., Brown J., Liu R.H. (2002). Antioxidant and antiproliferative activities of raspberries. J. Agric. Food Chem..

[24] Sousa M.S.B., Vieira L.M. (2011). Total phenolics and *in vitro* antioxidant capacity of tropical fruit pulp wastes. Braz. J. Food Technol..

[25] Zhishen J., Mengcheng T., Jianming W. (1999). The determination of flavonoid contents in mulberry and their scavenging effects on superoxide radicals. Food Chem..

[26] Rockenbach I.I., Rodrigues E., Gonzaga L.V., Caliari V., Genovese M.I., Gonçalves A.E.S.S., Fett R. (2011). Phenolic compounds content and antioxidant activity in pomace from selected red grapes (*Vitis vinifera* L. and *Vitis labrusca* L.) widely produced in Brazil. Food Chem..

[27] Sariburun E., Sahin S., Demir C., Turkben C., Uylaser V. (2010). Phenolic content and antioxidant activity of raspberry cultivars. J. Food Sci..

[28] Sousa S., Pinto J., Pereira C., Malcata F.X., Pacheco M.T.B., Gomes A.M., Pintado M. (2015). *In vitro* evaluation of yacon (*Smallanthus sonchifolius*) tuber flour prebiotic potential. Food Bioprod. Process..

[29] Herigstad B., Hamilton M., Heersink J. (2001). How to optimize the drop plate method for enumerating bacteria. J. Microbiol. Methods.

[30] Carrillo M.G., Ferrario M., Guerrero S. (2018). Effectiveness of UV-C light assisted by mild heat on *Saccharomyces cerevisiae* KE 162 inactivation in carrot-orange juice blend studied by flow cytometry and transmission electron microscopy. Food Microbiol..

[31] Kim D.K., Kim S.J., Kang D.H. (2017). Bactericidal effect of 266 to 279 nm wavelength UVC-LEDs for inactivation of Gram positive and Gram negative foodborne pathogenic bacteria and yeasts. Food Res. Int..

[32] Silva F., Ferreira S., Queiroz J.A., Domingues F.C. (2011). Coriander (*Coriandrum sativum* L.) essential oil: Its antibacterial activity and mode of action evaluated by flow cytometry. J. Med. Microbiol..

[33] Crowe J.H., Carpenter J.F., Crowe L.M. (1998). The role of vitrification in anhydrobiosis. Annu. Rev. Physiol..

[34] Gonzalez-Aguilar G., Robles-Sanchez R., Martinez-Tellez M., Olivas G., Alvarez-Parrilla E., de la Rosa L. (2008). Bioactive compounds in fruits: Health benefits and effect of storage conditions. Postharvest Stewart Rev..

[35] Champagne C.P., Møllgaard H., Farnworth E.R. (2008). Production of Probiotic Cultures and Their Addition in Fermented Foods. Handbook of Fermented Functional Foods.

[36] Champagne C.P., Mondou F., Raymond Y., Roy D. (1996). Effect of polymers and storage temperature on the stability of freeze-dried lactic acid bacteria. Food Res. Int..

